# Consultation on kidney stones, Copenhagen 2019: aspects of intracorporeal lithotripsy in flexible ureterorenoscopy

**DOI:** 10.1007/s00345-020-03481-9

**Published:** 2020-10-16

**Authors:** Søren Kissow Lildal, Kim Hovgaard Andreassen, Joyce Baard, Marianne Brehmer, Matthew Bultitude, Ylva Eriksson, Khurshid R. Ghani, Helene Jung, Guido Kamphuis, Peter Kronenberg, Ben Turney, Olivier Traxer, Øyvind Ulvik, Palle Jörn Sloth Osther

**Affiliations:** 1 Department of Urology, Aarhus University Hospital, Aarhus, Denmark; 2 Department of Urology, Herlev-Gentofte Hospital, University of Copenhagen, Copenhagen, Denmark; 3 Department of Urology, Amsterdam UMC, University of Amsterdam, Amsterdam, The Netherlands; 4 Department of Surgery and Urology, Danderyd University Hospital, Stockholm, Sweden; 5 Department of Clinical Sciences, Karolinska Institutet, Stockholm, Sweden; 6 Urology Centre and Stone Unit, Guy’s and St. Thomas’ NHS Foundation Trust, London, UK; 7 Department of Urology, University of Michigan, Ann Arbor, MI USA; 8 Department of Urology, Urological Research Center, Lillebaelt Hospital, University of Southern Denmark, Vejle, Denmark; 9 Department of Urology, Hospital CUF Descobertas, Lisbon, Portugal; 10 Department of Urology, Churchill Hospital, Oxford University Hospitals NHS Trust, Oxford, UK; 11 Department of Urology, Tenon Hospital, Paris, France; 12 Department of Urology, Haukeland University Hospital, Bergen, Norway

**Keywords:** Laser lithotripsy, Ureteroscopy, Kidney stones, Endoscopes, Safety, Technology

## Abstract

**Purpose:**

To summarize current knowledge on intracorporeal laser lithotripsy in flexible ureterorenoscopy (fURS), regarding basics of laser lithotripsy, technical aspects, stone clearance, lithotripsy strategies, laser technologies, endoscopes, and safety.

**Methods:**

A scoping review approach was applied to search literature in PubMed, EMBASE, and Web of Science. Consensus was reached through discussions at the Consultation on Kidney Stones held in September 2019 in Copenhagen, Denmark.

**Results and conclusions:**

Lasers are widely used for lithotripsy during fURS. The Holmium laser is still the predominant technology, and specific settings for dusting and fragmenting have evolved, which has expanded the role of fURS in stone management. Pulse modulation can increase stone ablation efficacy, possibly by minimizing stone retropulsion. Thulium fibre laser was recently introduced, and this technology may improve laser lithotripsy efficiency. Small fibres give better irrigation, accessibility, and efficiency. To achieve optimal results, laser settings should be adjusted for the individual stone. There is no consensus whether the fragmentation and basketing strategy is preferable to the dusting strategy for increasing stone-free rate. On the contrary, different stone scenarios call for different lithotripsy approaches. Furthermore, for large stone burdens, all laser settings and lithotripsy strategies must be applied to achieve optimal results. Technology for removing dust from the kidney should be in focus in future research and development. Safety concerns about fURS laser lithotripsy include high intrarenal pressures and temperatures, and measures to reduce both those aspects must be taken to avoid complications. Technology to control these parameters should be targeted in further studies.

## Introduction

Flexible ureterorenoscopy (fURS) has evolved as one of the major modalities for upper urinary tract stone management. This development is largely the result of continuing advancements in laser technology for intracorporeal lithotripsy, which is the focus of this review. Most daily clinical practices in the field of kidney stones, including lithotripsy techniques, are not supported by randomized trials and meta-analyses. This is not due to low research activity in this area, but rather to difficulties in designing meaningful trials that reflect daily clinical practice, because stone disease is so diverse. In this context, it is particularly important for practising experts to share their knowledge on clinical applications. The concept of the meeting “Consultation on Kidney Stones”, on which this review is based, was to create a forum for transfer and development of clinical expertise.

## Methods

To evaluate lithotripsy in fURS regarding the balance between existing evidence, expert opinions, and safety and efficacy of new technological improvements, key opinion leaders in the field were invited to assess and discuss existing evidence at the 2-day meeting entitled “Consultation on Kidney Stones: Aspects of Intracorporeal Lithotripsy” held in Copenhagen, Denmark, in September 2019. The participating experts were assigned different topics and prepared presentations according to scoping reviews using PubMed, EMBASE, and Web of Science to search the literature. The first day of the meeting was open only to the experts, who individually presented their topics, which were then discussed by the whole group. The presentations were subsequently adjusted if necessary. The second day was an open meeting at which all presentations were given to an international audience, and this was followed by free discussions.

## Basics of laser lithotripsy

### Laser fibre size

The deflection range of a flexible ureteroscope decreases with increased size of the laser fibre inserted [1–3]. The size of the fibre also has an inverse effect on irrigation [1, 2]. An in vitro study of the ablation efficiency of laser fibres showed that large fibres (550 μm) produced wider fissures than small fibres (200 μm), but the fissures made by the small fibres were deeper [4]. Another investigation found that using a larger fibre created more retropulsion of the stone during lithotripsy [5]. Comparison of total fragmentation volume indicated little difference between fibres except at very low pulse energies (0.2 J), at which small fibres were more efficient [4]. These observations suggest that smaller fibres provide the following: better irrigation and thus improved visibility; better deflection and hence increased accessibility; and less retropulsion and thus higher ablation efficiency, and also an overall reduction in operating time [2, 6].

### Laser fibre tips

Laser fibres can have a standard flat tip or a polished or ball-shaped tip. The ball-tip fibre is designed to reduce damaging friction forces generated within the working channel of the ureteroscope. It has been shown that this type of fibre can be safely passed through a deflected ureteroscope without causing liner perforation. The same advantage can be achieved by cleaving a standard fibre in such a manner that the coating protects the scope from the laser core [7]. Flat-tip fibres require greater insertion force at all angles and therefore can cause the ureteroscope liner to leak if it is deflected 45°or more [8].

An in vitro study comparing lithotripsy performance of different types of laser fibres found that the standard fibre functioned just as well as specially designed fibres [9]. Kronenberg et al. [10] noted that, compared to fibres stripped off the coating material, coated fibres achieved significantly higher ablation volumes, and there was no difference in performance between coated fibres cleaved with metal or ceramic scissors. However, Aldoukhi et al. [11] observed that single-use fibres and cleaved reusable fibres performed better than fibres with tips cut using Mayor scissors. The difference in efficiency between stripped and coated fibres may be due to the fibre itself being damaged during the stripping off the coating, leading to a dispersion of laser energy [10, 11]. Another downside of such stripping is that a fibre devoid of its coloured coating is difficult to discern in front of the ureteroscope. The above-mentioned factors have not been scientifically evaluated in vivo, and individual surgeons may have different preferences.

### Laser settings

Various laser systems have been developed that offer different laser settings, such as high frequency and long-pulse duration. The total power (W) used is equal to the pulse energy (J) multiplied by the pulse frequency (Hz). In an in vitro study, a combination of low frequency and high pulse energy was more effective than the opposite combination with the same total power level, and pulse energy and ablation volume were linearly correlated with the width and depth of fissures observed in the stone material [4]. However, high pulse energy resulted in more stone retropulsion, larger stone fragments, and laser fibre burn-back, all leading to prolonged operating time [4, 12]. Lowering the pulse energy and increasing the frequency give rise to less retropulsion and fibre burn-back, which results in dusting of the stone. Regarding the pulse length, preliminary data from an in vitro study showed that short pulse settings led to significantly higher ablation volumes than long-pulse settings [13], whereas long-pulse lithotripsy was associated with less fibre burn-back, smaller fragments, and more dust. Other investigations have been unable to demonstrate a significant relationship between pulse duration and stone ablation [14, 15].

The Moses technology introduces a pulse-shaped modulation that optimizes energy delivery through water to the target stone. The Moses platform has two settings: Moses contact (MC) mode, for operation at close range (< 2 mm); Moses distance (MD) mode, for lithotripsy at a distance of 2 mm. In a preclinical study, the Moses technology resulted in more efficient laser lithotripsy with significantly reduced stone retropulsion [16]. In an automated in vitro dusting model, compared to long-pulse lithotripsy, Moses holmium:yttrium–aluminum–garnet (Ho:YAG) (Ho:YAG) laser technology provided greater ablation of soft stones when in contact with the stone surface [17]. In an investigation using a three-dimensional positioning system, researchers examined the impact of laser fibre working distance on fragmentation when altering pulse width or modulation [18], which showed that holmium laser lithotripsy was significantly affected by the fibre tip to stone working distance, with the greatest ablation volume obtained with the fibre in contact with the stone.

Considering optimal settings for the lithotriptor, different stone compositions require different settings to achieve the desired effect, and the desired effect may vary in individual situations. The recommendation is to start with a test setting of low pulse energy and low frequency to initially determine how the stone reacts, and thereafter adjust settings accordingly to improve efficiency of the lithotripsy.

## Technical aspects of laser lithotripsy and stone clearance during fURS

Various methods for laser lithotripsy have been described, and combining them can lead to more efficient ablation and clearance of stones with different characteristics [19]. The techniques described include dusting (dancing/painting), chipping, fragmentation, popcorning, dustmenting, and popdusting, and all these strategies can be combined in different ways to achieve the desired outcome. Dancing/painting is done by moving the laser fibre from side to side over the surface of the stone, using dusting settings. Chipping entails lasering off small pieces from the edges of the stone leaving small fragments for spontaneous passage, whereas fragmenting involves cutting a stone into larger fragments for subsequent basket removal. The fragmentation and basketing strategy use a setting of low frequency (4–10 Hz) and high pulse energy (0.6–2.0 J); dusting uses a setting of high frequency (15–80 Hz) and low pulse energy (0.2–0.5 J); popcorning stones requires settings of moderate-to-high frequencies (10–20 [40] Hz) and moderate pulse energy (1–1.5 J)[20–23].

Popcorning refers to a non-contact technique in which the laser energy whirls around fragments that come in contact with the laser and breaks them into smaller pieces. Chawla et al. [20] conducted an in vitro investigation of popcorning with different laser settings and observed that 1.5 J and 40 Hz produced the greatest mean decrease in stone burden per amount of time used; however, such a high-power level cannot be recommended in vivo. In that study, settings of 1.0 J and 20 Hz were most efficient with regard to mean stone weight loss per total amount of energy used. Emiliani et al. [24] suggested that a good compromise for popcorning would be using a long pulse of 1.5 J and 20 Hz with a 270 μm laser fibre and taking as much time as possible (> 4 min) to produce clinically insignificant fragments. In another in vitro model, Aldoukhi et al. found that the popcorn te

chnique was more effective when the laser fibre was positioned in contact with the stone as compared to at a distance of 2 mm from the stone, and when performed in a small (11 mm) rather than a larger calyceal model [25]. These in vitro studies do not replicate the clinical environment, and, working in a limited space, surgeons should always aim to choose a safe power level based on the irrigation flow applied and other factors, including outflow (access sheath) and temperature of the irrigation fluid.

Humphreys et al. [26] conducted a non-randomized prospective clinical comparative trial considering dusting versus fragmenting, and they found no statistically significant difference in stone-free rate (SFR) and no difference in complication rates between the two patient groups. However, although stones were significantly larger in the dusting arm (96 vs 63 mm^2^, *p* < 0.001) the mean operative time was significantly shorter in that group (36 vs 67 min, *p* < 0.001). This agrees with clinical data showing that, for large stones, it is easier to control the complete stone burden during dusting, whereas fragmenting may result in fragments moving into multiple different calyces in which they must be dealt with, potentially prolonging OR time and reducing SFR.

Ureteroscopic lasering of lower pole stones can be challenging. The anatomy of the lower pole must be considered, because a long infundibular length, a narrow infundibular width, and a steep infundibulo-pelvic angle may have a negative impact on stone clearance, especially if more than one of these aspects is unfavourable [27–30]. Using a basket/grasper to move the stone to another location can be a viable alternative that can facilitate lithotripsy, reduce scope damage, and aid fragment passage [31, 32]. Schuster et al. [33] compared SFR in patients with lower pole stones treated in situ and subjects with relocation of the stone before lithotripsy. For stones > 1 cm in size, complete clearance was obtained in 100% of cases by relocation versus 29% of cases in situ (*p* = 0.005); the difference between the two groups was not significant for stones of < 1 cm. Placing the patient in Trendelenburg position during surgery can facilitate stone relocation to the upper pole from other sites by creating a dependent upper pole; two randomized trials have demonstrated that this method results in shorter OR time, better SFR, less flexible ureteroscope manipulation, and less stone migration into the lower pole [34, 35]. If the stone cannot be relocated due to size or anatomy, or if lithotripsy in situ is preferred, a ball-tip laser fibre can be safely introduced through a deflected scope.

The 2020 EAU Urolithiasis Guidelines recommend that only baskets made of Nitinol be used for fURS. A range of different baskets are available, such as triangular baskets, 4-wire baskets, smaller baskets for narrow locations, and larger baskets for strength. Baskets can also vary regarding penetration force, radial dilation force, and opening dynamics, and add different degrees of resistance to scope deflection [36]. Bach et al. [37] tested various sizes of baskets in five different endoscopes and found that, in contrast to laser fibres, the size of the basket did not influence the deflection of the scope, which made relocation of a stone easier than in in situ lithotripsy. However, the size of the basket had an inverse effect on irrigation flow. Conversely, Patel et al. [36] showed some limitation of deflection, which was greater for a 2.2 Fr compared to a 1.5 Fr basket. In practice, these differences will vary depending on the type of ureteroscope employed and how long it has been in use, and the specific basket that is chosen.

Methods for the evacuation of small residual fragments include the glue-clot technique and irrigation/suction systems. In the glue-clot technique, autologous venous blood is injected into a calyx, where it agglutinates with stone fragments to form a clot that can be extracted with a basket [38]. In a randomized pilot study of 47 patients, an automated irrigation/suction pump system was tested and found to reduce mean operating time by 35% (*p* = 0.04) and to increase SFR from 69 to 92% (*p* = 0.048) compared with the standard pressurized technique [39].

## Comparing clinical outcome of different laser lithotripsy strategies

Comparing fragmenting and dusting with regard to clinical outcomes has proven difficult, because the objective metrics for such comparison vary. The studies performed have differed regarding rates of pre-stenting and staged procedures, and ways of reporting complication grades, emergency room visits, intensive care admission, hospitalization, and re-intervention. Reports concerning follow-up also differ with regard to definitions of stone free, imaging modalities, and timing of the follow-up and repeat intervention rate. Stone free has been defined as zero fragment, < 2 mm, or > 4 mm residual fragment. Imaging modality for follow-up varies between computed tomography (CT), kidney–ureter–bladder radiograph (KUB), ultrasound (US), or fluoroscopy at operation, and a large proportion of patients apparently have no follow-up imaging at all [40]. An assessment of administrative data in the United States showed that 39% of patients had no imaging at 12 months post-surgery[41].

A retrospective study demonstrated that fragments > 4 mm in size were associated with significantly higher rates of stone growth leading to complications and need for re-intervention, whereas fragments of > 2 mm were likely to grow, but were not associated with complications or re-intervention [42]. Another retrospective investigation reported that the cumulative repeat surgery rate at 5 years after fURS was proportional to the size of residual fragments on post-operative CT [43]: with no visible residual fragments, fragments < 4 mm, or fragments > 4 mm, the rates were 3.5%, 8.2%, and 46.2%, respectively. CT has been reported to have a sensitivity of 95–100% for residual fragments [44].

Eleven studies comparing spontaneous passage and fragmentation with active retrieval [45] were identified, but most of them could not be compared with each other due to differences in imaging modalities and definitions of SFR. Only three comparative, non-randomized studies of the various disintegration strategies in the treatment of renal stones were pinpointed (Table 1) [26, 46, 47]. Two of those assessments used CT for follow-up, whereas the third used KUB and US. Only one of those three studies reported the power settings for dusting and fragmentation, respectively.

**Table 1:** Comparative studies regarding SFR with spontaneous passage vs active retrieval during lithotripsy

Cohort	Lee et al. 2016		Humphreys et al. 2018		El-Nahas et al. 2019	
	Spontaneous	Retrieval	Spontaneous	Retrieval	Spontaneous	Retrieval
Study design	Retrospective	Retrospective	Prospective	Prospective	Retrospective	Retrospective
Study period	2010–2015	2010–2015	2013–2016	2013–2016	2015–2017	2015–2017
Cases, *n*	76	172	68	82	51	56
Mean stone size (SD)	11 mm (± 5.2)	11 mm (± 4.8)	11 mm (± 4.3)	8.8 mm (± 3.5)	NS	NS
Laser power (setting)	NS	NS	30–100 W (NS)	30–100 W (NS)	20–60 W (0.3 J/20 Hz)	20–60 W (1.0 J/10 Hz)
OR time	82 min	83 min	36 min	67 min	76 min	91 min
Stenting rate	100%	100%	100%	100%	100%	100%
Complications	11%*	11%*	13.2%*	19.5%**	8%*	9%**
Definition of SFR	< 3 mm	< 3 mm	No fragment	No fragment	No fragment and < 4 mm	No fragment and < 4 mm
Imaging used	CT	CT	KUB, US	KUB, US	CT	CT
Imaging timing	4 weeks	4 weeks	4–6 weeks	4–6 weeks	2 months	2 months
Stone free rate	87%	89%	58%	74%	59% and 86%	79% and 89%

*SD* standard deviation, *OR* operating room, *SFR* stone-free rate, *CT* computed tomography, *KUB* kidney–ureter–bladder radiograph, *US* ultrasound

In a randomized, double-blinded clinical trial comparing regular and Moses modes of Ho:YAG laser lithotripsy, Ibrahim et al. showed that the Moses technology was associated with significantly lower fragmentation/pulverization (stone dusting) and procedural times, which the authors explained by less retropulsion of stones during Moses mode lithotripsy [48].

Conclusive evidence for comparing spontaneous passage and active retrieval strategies is limited, and it is difficult to compare existing studies, because they vary regarding disintegration strategy, SFR criteria, and time and modality of imaging at follow-up. Moreover, the investigations have not systematically reported stone density and composition and post-operative complications.

## Endoscopes and laser lithotripsy

**Fig. 1:**
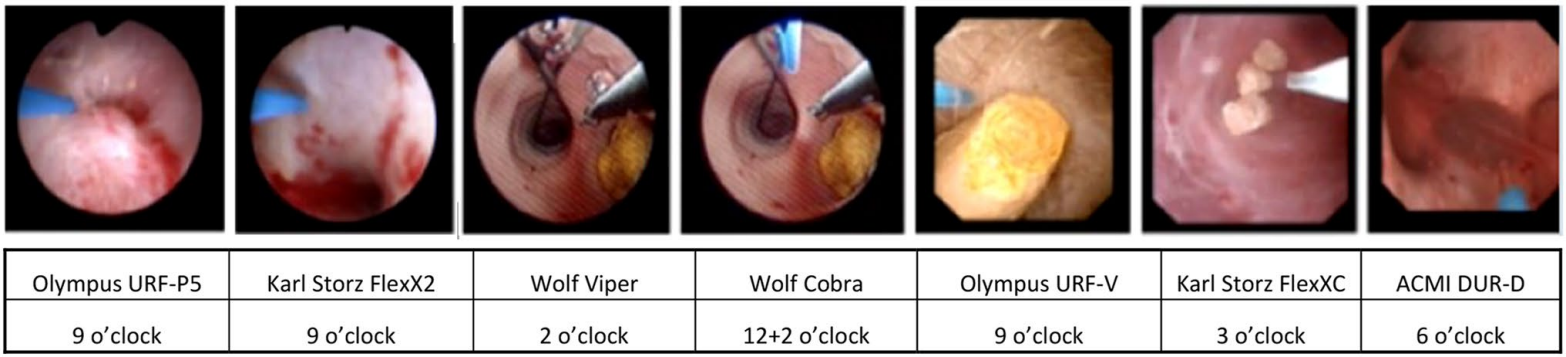
Different positions of working channel depending on manufacturer and model of ureteroscope

Ureteroscopes vary with respect to location of working channel (Fig. 1), deflection ability and irrigation flow with laser fibre inserted, distance of laser tip from the tip of the endoscope, and optical imaging quality and illumination of the surgical field. All of the listed features have a potential impact on laser lithotripsy, although evidence for this is based mainly on in vitro studies and expert opinions, and very little has been published regarding effects on clinical outcome.

The position of the working channel of the endoscope can influence irrigation flow and thus create differences in the flushing and movement of stone and tissue. The position also dictates location of the laser fibre/basket inserted through the channel and thereby affects the ability to reach stones in certain positions within the kidney [49].

In vitro comparison of single-use and reusable flexible ureteroscopes showed that the latter had bettervision characteristics [50]. It appears that scopes differ regarding occurrence of light flashes during laser lithotripsy, over-illumination of the field, and the speed at which light intensity can be regulated. These phenomena may occur due to automatic light intensity regulation being affected by laser energy flashes or by stone fragments or dust that is flushed around by irrigation and laser pulse pressure waves. The cited in vitro comparison also demonstrated that the flow varied between different flexible ureteroscopes equipped with the same type of laser fibre [50]. The problem of working channel damage can be almost completely overcome by employing single-use flexible ureteroscopes.

## Safety aspects of laser lithotripsy during fURS

Direct contact of the laser can cause local burns on the urothelium. Furthermore, high-flow irrigation for optimal visualization and cooling leads to increased intraluminal pressure, although such irrigation is necessary.

It should also be noted that incorrect handling of lasers may damage the ureteroscope and even harm the surgeon and OR personnel. A safety distance between the tip of the fibre and the tip of the scope should be maintained to minimize the risk of damaging endoscopes. Talso et al. [51] observed that the length of the fibre projecting from ureteroscopes was 1–2 mm at first appearance in the camera image, whereas it was 3–4 mm when reaching one-fourth of the screen, and in that position, the laser bubble was never in contact with the tip of the ureteroscope and was therefore protected from damage.

The incidence of ureteral perforation during ureterorenoscopy has been reported to be 0.4–6.3%, and the risk factors identified included size of ureteroscope, prolonged operation time, surgeon’s lack of experience, stone characteristics, lack of pre-stenting, and high laser energy [52, 53]. Possible complications of perforation are extravasation, haematoma, sepsis, pain, obstruction, stricture, and loss of kidney function [52–55]. Use of laser during intracorporeal lithotripsy has been observed to be an independent risk factor, with a 3.6% risk of Post-ureteroscopic Lesion Scale (PULS; [56] grade 1 lesions and 3.1% risk of PULS grade 2 lesions. To avoid ureteral perforation, it is recommended that optimal visualization be secured by irrigation and that a safety distance of at least 1 mm be maintained between laser tip and urothelium.

During laser lithotripsy, visualization can be impaired by stone dust and debris. Increasing irrigation to solve this problem unequivocally raises the intrarenal pressure, potentially leading to tubular, venous, and lymphatic backflow. Baseline intrarenal pressure is approximately 10 mmHg, and the threshold for backflow is 30–35 mmHg. Intrarenal pressure levels during fURS and holmium laser usage have been noted to be as high as 50–350 mmHg [57, 58]. Intrarenal pressures above 30 mmHg for more than 10 min have been shown to significantly increase the incidence of septicaemia in percutaneous nephrolithotomy [59]. Although several studies have suggested that complications such as sepsis and post-operative pain leading to longer hospitalization are related to high intrarenal pressure, no definitive causal data exist [60–63]. Intrarenal pressure during fURS may be reduced by the use of a ureteral access sheath (UAS) [64]. If a UAS is to be applied, it must have a larger diameter than the scope, which make the UAS a double-edged sword: on one hand, it reduces intrarenal pressure and potential septic complications, and on the other hand, it may increase the risk of strain-induced lesions to the ureter [65]. Therefore, it is important to use the smallest UAS compatible with the ureteroscope chosen to ensure both safe placement without significant friction and sufficient outflow, and thereby achieve both low intrarenal pressure and sufficient flow for visualization during lithotripsy [66].

High intrarenal temperatures have been observed during laser lithotripsy, especially at high total power settings (> 30 W) [67]. High temperatures can lead to thermal ablation of kidney tissue, resulting in permanent kidney damage. Overall, such tissue damage is dependent on temperature, time, and blood flow [68]. In the ureter, the threshold for tissue damage has been shown to be around 43 °C for 120 min, which is equivalent to approximately 50 °C for 56 s or 56 °C for 0.9 s [69–71]. In an in vitro model, Wollin et al. achieved temperatures of > 43 °C at all laser settings within 1 min without irrigation, and the maximum temperature reached was 100 °C at fragmentation settings [69]. Aldoukhi et al. recorded temperatures in a pig kidney during high-power holmium laser lithotripsy at 0.5 J and 80 Hz and different irrigation flows, and found that only high irrigation flow (38–40 ml/min) could keep the caliceal temperature at ≤ 43 °C [72]. To reduce heating, it is important to have secured continuous flow irrigation and consider use of room temperature or even cooled irrigation fluid. Lasering should be carried out intermittently to allow cooling in low-flow areas.

## Comparison of different laser technologies for intracorporeal lithotripsy

The past and current standard for laser lithotripsy is the Ho:YAG laser, which has revolutionized retrograde stone management. Introduction of high-power Ho:YAG laser systems that utilize laser settings of high frequency and low pulse energy (HiFr-LoPE), varying pulse lengths, and Moses technology for reduction of retropulsion and improvement of fragmentation have expanded the surgeon’s ability to ablate urinary tract stones and thereby opened a new era for the dusting technique. Reducing the need for fragment retrieval and the potential hazards of this technique (UAS usage and multiple scope passages) may be advantageous [73]. Recently, a new laser technology for stone management has emerged—the thulium fibre laser (TFL). The TFL emits light with a wavelength of 1940 nm, compared to 2100 nm with Ho:YAG [74–79]. Comparing laser setting options, TFL offers frequencies of 5–2200 Hz, pulse energies of 0.025–6 J, and pulse durations of 200–12,000 microsec [76, 80–83], whereas Ho:YAG offers 5–100 Hz, 0.2–6 J, and 50–1300 microsec [81, 84]. These qualities of the TFL laser give this technology the potential to produce 3–4 times more dust compared to high-power Ho-YAG lasers [78, 85, 86]. Furthermore, due to the novel laser beam generation by laser diodes in TFL, smaller fibres (50 µm) can be utilized, which may result in higher energy intensity at the tip of the fibre, better scope deflection, and better irrigation, all of which can potentially increase stone ablation volume at comparable settings [74, 80, 81]. Initial reports of TFL performance in the clinical setting are promising but still limited [87], and further evaluation, including consideration of safety aspects (temperature) and ability of the laser to embrace all lithotripsy strategies, will be necessary to define the role of TFL. To date, no clinical comparative studies of holmium and thulium fibre lasers have been conducted. Nevertheless, both the HiFr-LoPE Ho:YAG and the new TFL technology show promise in expanding the role of flexible ureteroscopic stone management by approaching larger stones and more complex stone scenarios.

## Conclusions

Lasers have revolutionized the scenario of intracorporeal lithotripsy during fURS. Ho:YAG is still the predominant laser for stone management, and, with this technology, specific settings for dusting and fragmenting have evolved, which have expanded the role of fURS in endoscopic stone management. Recently, the TFL was introduced, and this new laser source may prove to further increase the efficiency of laser lithotripsy. There is still debate as to whether the fragmentation and basketing strategy are preferable to the dusting strategy for increasing SFR. However, one does not exclude the other, because different stone scenarios call for different lithotripsy approaches, and, for large stone burdens, all laser lithotripsy settings (dusting, fragmenting, and popcorning) may often be necessary for optimizing the final result. Technology for removing dust from the kidney should be a focus for future research and development. Safety aspects of fURS laser lithotripsy include high intrarenal pressures and temperatures. Inasmuch as measures to reduce both pressure and temperature are mandatory to avoid sepsis, bleeding, and nephron loss, technology to control these parameters should be targeted in further investigations.
